# Supplementary material for: Loss of transcriptional plasticity but sustained adaptive capacity after adaptation to global change conditions in a marine copepod
Source: Nat Commun. 2022 Mar 3;13:1147. doi: 10.1038/s41467-022-28742-6 (PMC8894427; doi:10.1038/s41467-022-28742-6)
Supplement: Supplementary file 1 — Supplementary information [file 41467_2022_28742_MOESM1_ESM.pdf]

Supplementary Methods

Validation of RNA-based allele frequency estimates

Because our allele frequency estimates were derived from RNA, it is possible that allele specific expression (ASE) could influence these estimates. We thoroughly explore the possible impacts of this phenomenon on our results by 1) presenting new data to quantify the degree of correlation between allele frequency estimates between pools of DNA and RNA in this study; 2) comparing the global (Figs. 2, S1, S4) and locus specific patterns of gene expression and allele frequency estimates; 3) investigating the loss of genetic diversity following transplant; and 4) considering the literature of ASE within populations and the impact of low levels of linkage disequilibrium in *Acartia tonsa* on bias due to ASE. These analyses, below, give confidence that our allele frequency estimates from RNA are comparable with those derived from DNA.

We recently published results from a related experiment that utilized the same non-transplanted lines as in this manuscript (AM_{AM}, OWA_{OWA}) at F25 and included allele frequency estimates from pools of DNA [1]. We compared the allele frequency estimates derived from RNA at F3 (equivalent to F23) to those from DNA at F25. These two datasets overlapped at 6,983 variant loci, of which 228 were consistently divergent between AM_{AM} and OWA_{OWA} (see details in main text for how these were identified). We directly compare these allele frequency estimates in figure S5. The estimates were highly congruent with R^2 values of 0.83. We next distinguished the drivers of the differences in these allele frequency estimates between DNA and RNA. Here, the variation could be driven by 1) allele specific expression; 2) drift from generation F23 to F25; 3) technical variation from sampling individuals for sequencing; 4) technical variation from finite sequencing depth. We were interested in determining if the variation between DNA and RNA could be due to drift and technical variation without any ASE. To determine the impact of these different factors, we simulated 2000 datasets where the observed DNA allele frequency estimates were treated as the true values. For each replicate, we simulated two generations of evolution assuming under the Wright-Fisher model assuming no linkage and with parameters matching our experimental design. We also included technical variation in pooling individuals for sequencing as well as finite coverage.

These simulations show ASE is playing a minimal role in the differences in allele frequency estimates between data derived from pooled DNA and RNA. The variance in allele frequency estimates from technical causes explains almost all the differences in allele frequency estimates between the two data sources (Fig. S5). Looking at the differences in allele frequency estimates between DNA and RNA, the 95% confidence intervals from the simulated data overlap with almost the entire distribution of observed differences in the estimates. This shows that ASE is not a major driver in the variation between the two data sources. However, there are a small fraction of loci that show extreme differences between our observed DNA vs. RNA estimates that are not replicated in the simulated data; these differences could be driven by ASE. Despite this, none of the divergent loci between AM and OWA were outside of the simulated distribution of allele frequencies, providing further evidence that ASE is not driving the differences in allele frequencies between the lines.

We next explored global patterns in the relationship between allele frequency estimates and gene expression. Overall, we found no relationship between gene expression and allele frequency divergence as demonstrated in Fig. S1 and S4. The PCA shows that gene expression data are relatively noisy (Fig. S1). In contrast, the divergence in allele frequency is quite clean and consistent between AM and OWA (For example, see the allele frequency PCA along PC 1, Fig. S1). Similarly, there is almost no relationship between DGE and allele frequency divergence (Fig. S4). Given the limited relationship between the DGE and allele frequency estimates, it is unlikely that ASE would be entirely driving this striking divergence between OWA and AM, particularly given the relative noise in gene expression data.

The changes in genetic diversity following transplant also indicate that genetic divergence, rather than ASE, are driving the observed allele frequency changes. We found a loss of genetic diversity in regions of the genome that were divergent in allele frequency between AM and OWA in OWA_{AM} but not AM_{OWA} lines. If the signal of divergence between AM and OWA was driven by allele specific expression, we would expect to observe consistent signals in both AM_{OWA} and OWA_{AM}. However, we only see a signal in shifts in diversity for OWA_{AM}, indicating that these changes were driven by genetic changes rather than ASE.

It is known that ASE is prevalent within individuals and populations, yet our analysis here indicates allele frequency estimates can be accurate despite this phenomenon. While a number of studies have shown that genotype calls from RNAseq of individuals are accurate [2], only a few studies have looked at the accuracy of using pooled RNAseq data to determine allele frequencies. These pooled studies have found that, in agreement with our results, estimates derived from RNA are on par with pooled DNA estimates [3]. Studies of ASE in copepods hybrids among diverged mitochondrial lineages [4], in humans [5,6], and plants [7], suggest that while 30% of transcripts may be affected by cis-regulatory variants, most differences in allelic expression are less than a 60:40 ratio (refs above). For example, the average expression ratio is 50:50 with a narrow distribution even for copepod F1 hybrids (diverged *Tigriopus californicus* lineages) reared in a stressful, high temperature environment (see Fig 2B from Tangwancharoen et al. [4]). While any difference in allelic expression could affect allele frequency estimates from RNA, ASE is unlikely to affect global patterns of allele frequency estimates, which we show are very strongly and consistently different between lines.

The extent to which ASE will skew allele frequency estimates depends on the levels of linkage disequilibrium (LD) within the population [8]. Because ASE is due to cis regulatory variantion, only variants in linkage with this cis-variation will be biased in their frequency. As such, for populations with low levels of LD, estimates from pooled RNAseq can be accurate. In our data, LD decreases to background levels by about 200 base pairs (unpublished data). Given this rapid decay in LD, only a minority of any expressed transcripts will be influenced by ASE and the vast majority of our data will not be impacted by this phenomenon, as indicated by the results shown above.

Genome-wide variation in allele frequencies and gene expression following transplant

Additional analyses were run to validate the results observed in Fig. 4, where gene expression converged on the adaptive profile across generations, but allele frequencies shifted only in OWA_{AM}. We considered expression patterns in the home environment as the adaptive transcriptomic profile for that environment, achieved either through plastic or evolutionary mechanisms (x-axes of Fig. S5A). These adaptive transcriptional profiles were compared to the change in gene expression of a line in transplanted versus long-term selected conditions (y-axes

of Fig. S5A). This comparison of long- versus short-term responses across three successive generations allowed us to test if copepods transplanted to the opposite environment can match expression patterns to the long-term, adapted profile. A correlation between long-term, adapted expression (x-axis) and transplant expression change (y-axis) indicates the degree to which transplanted lines can match their expression to the optimal profile for the environmental condition, where a positive correlation represents adaptive changes in expression [9]. After one generation in transplant conditions, gene expression was positively correlated with the adaptive expression for both lines, AM transplanted to OWA and OWA transplanted to AM (Fig. S5A top panel; $\rho_{AM} = 0.57$, $\rho_{OWA} = 0.64$, $P < 0.001$) and this correlation increased each successive generation. Discriminant analysis of principal components (DAPC) showed the same convergence on the adaptive transcriptomic profile (Fig. S5). In accord, the proportion of positively correlated, adaptively expressed genes (dark points; Fig.S5A) increased across generations for both ambient and warming and acidification lines ($P < 0.05$; Proportions: $F1_{AM} = 0.85$, $F2_{AM} = 0.87$, $F3_{AM} = 0.90$, $F1_{OWA} = 0.66$, $F2_{OWA} = 0.94$, $F3_{OWA} = 0.95$) with the greatest response in warming and acidification relative to ambient lines, 29% versus 5% increase in the proportion adaptive. arriving at 90-95% adaptive gene expression profiles.

We assessed the degree of adaptive evolution for each transplanted line across the generations by quantifying the change in allele frequencies of adaptive loci in transplanted lines. We consider the adaptive difference in allele frequencies as the difference between mean frequencies of AM and OWA lines (for the significant loci identified with the CMH test across all generations) and assess the convergence of transplanted lines on this adaptive difference (i.e., do AM in OWA frequencies converge on the OWA allele frequency?). Spearman's correlation was used to determine the extent to which these frequencies were correlated. We compared the mean difference in allele frequency between OWA_{OWA} and AM_{AM} for 17,720 loci that had evolved consistent divergence in allele frequency (x-axes of Fig. S5B) to the mean difference in allele frequency in response to transplant conditions (OWA_{am} AM_{OWA}; OWA_{AM} AM_{OWA}; y-axes of Fig. S5B). OWA_{AM} copepods evolved to match allele frequencies of the ambient adaptive alleles with increasing changes in frequencies across each successive generation (Fig. S5B). In contrast, AM_{OWA} lines did not undergo the same rapid adaptation but maintained allele frequencies (Fig. S5B), even though their transcriptional profiles shifted to match warming and acidification

adaptive expression (Fig. 3A). Likewise, the proportion of adaptive alleles (dark points) increased across transplant generations for the warming and acidification lines, but not the ambient lines (Fig. 3B; proportions test: $P < 0.001$; Proportions: $F1_{AM} = 0.68$, $F2_{AM} = 0.64$, $F3_{AM} = 0.65$; $F1_{OWA} = 0.68$, $F2_{OWA} = 0.75$, $F3_{OWA} = 0.76$).

We realize that criticism has been leveraged against past work that has used similar analyses as in Fig. S5 where the x and y axes share a denominator are not independent [10,11]. This should lead to a positive correlation between the variables, as we observe here. Despite this, there is no reason to expect a consistent change in this correlation across generations as we observe for both the gene expression and allele frequency analysis. Therefore, while some proportion of the positive correlations we observe is due to statistical bias, the increase indicates that lines are shifting gene expression and allele frequency (in AM only) to match their transplanted environment, in agreement with the DAPC in the main text (Fig. 4).

Experimental considerations

We also acknowledge the presence of a degree of pseudoreplication in the design where each environmental condition is housed in a single incubator [12]. That is, some degree of the responses observed here may be due to specific incubator effects rather than experimental conditions. However, there has been criticism of the idea that shared spatial arrangements between treatments, such as a room or an incubator, necessarily result in statistical dependencies [13,14]. In our case, both CO_2 and temperature were held constant throughout the experiment, continuously monitored, and verified by independent measures (Table S1). We also know that 22 °C is 4 degrees beyond the optimum of 18 °C and results in significant mortality [15]. Finally, the transcriptomic and genomic effects we observe are consistent with both high temperature and low pH stress. Therefore, while we cannot completely rule out incubator specific effects, it is likely that the majority of the responses observed are due to the experimental treatments.

Supplementary Table 1: Environmental variables. Each temperature and CO_2 level used in this experiment was evaluated to ensure that environmental conditions were accurate. SD = standard deviation, N = number of observations, SE = standard error of the mean.

Environment	Variable	Target value	Mean measured value	SD	N	SE
Ambient	Temperature	18°C	18.16	0.463	64	0.058
warming and acidification	Temperature	22°C	21.89	0.402	60	0.052
Ambient	pH	8.2	8.35	0.093	64	0.011
warming and acidification	pH	7.5	7.61	0.099	60	0.012
Ambient	pCO_2	400µatm	380.94	49.30	9	16.43
warming and acidification	pCO_2	2000µatm	2358.1	120.81	9	40.27

Supplementary Figures

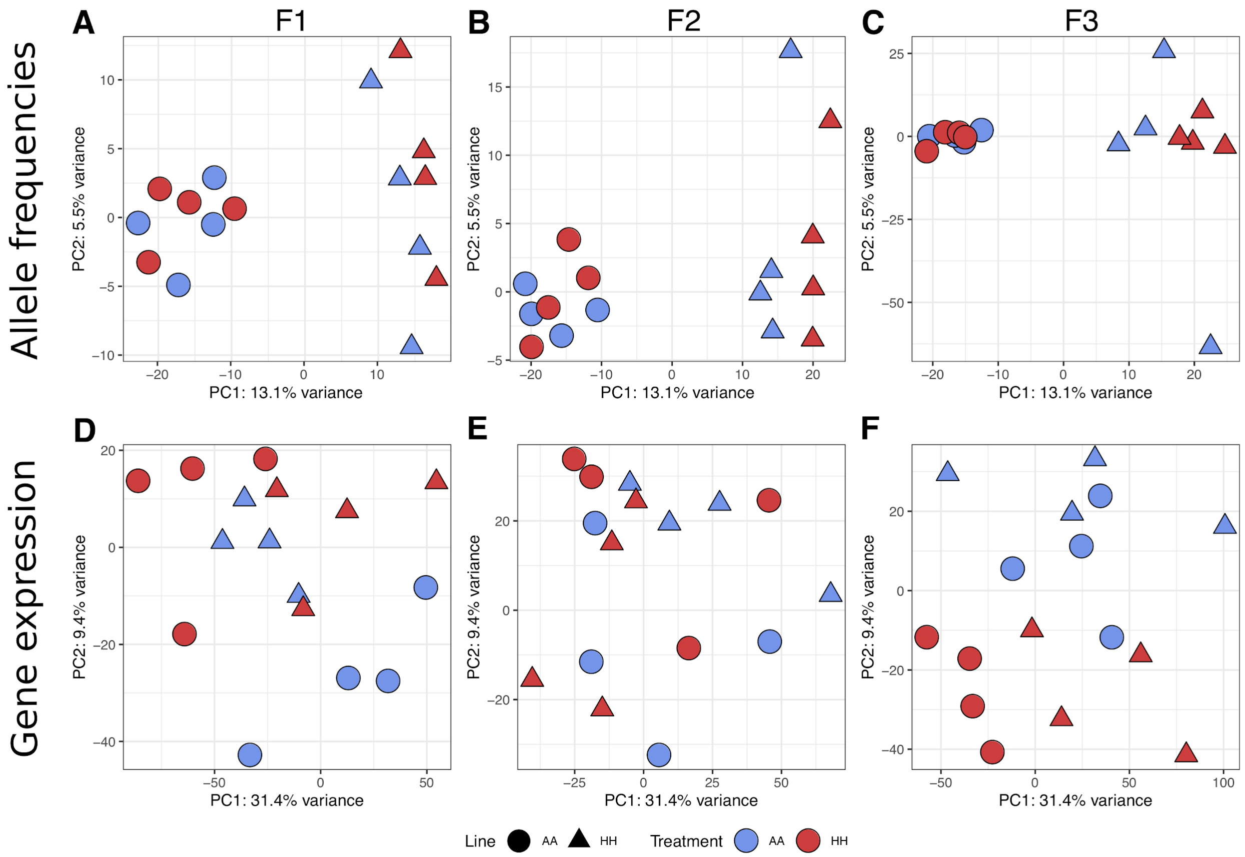

Figure S1: Principal component analysis of genome-wide genetic variation (panels A-C) and gene expression patterns (panels D-F) during three generations of reciprocal transplant following 20 generations of selection for ambient (AA) and warming and acidification (HH) conditions. The analysis here was conducted on all generations together. Shape indicates selection line and color indicates environmental condition at that generation. Both gene expression and genetic variation clustered by line, rather than condition at F1. By F3, a line effect was evident for both lines' gene expression.

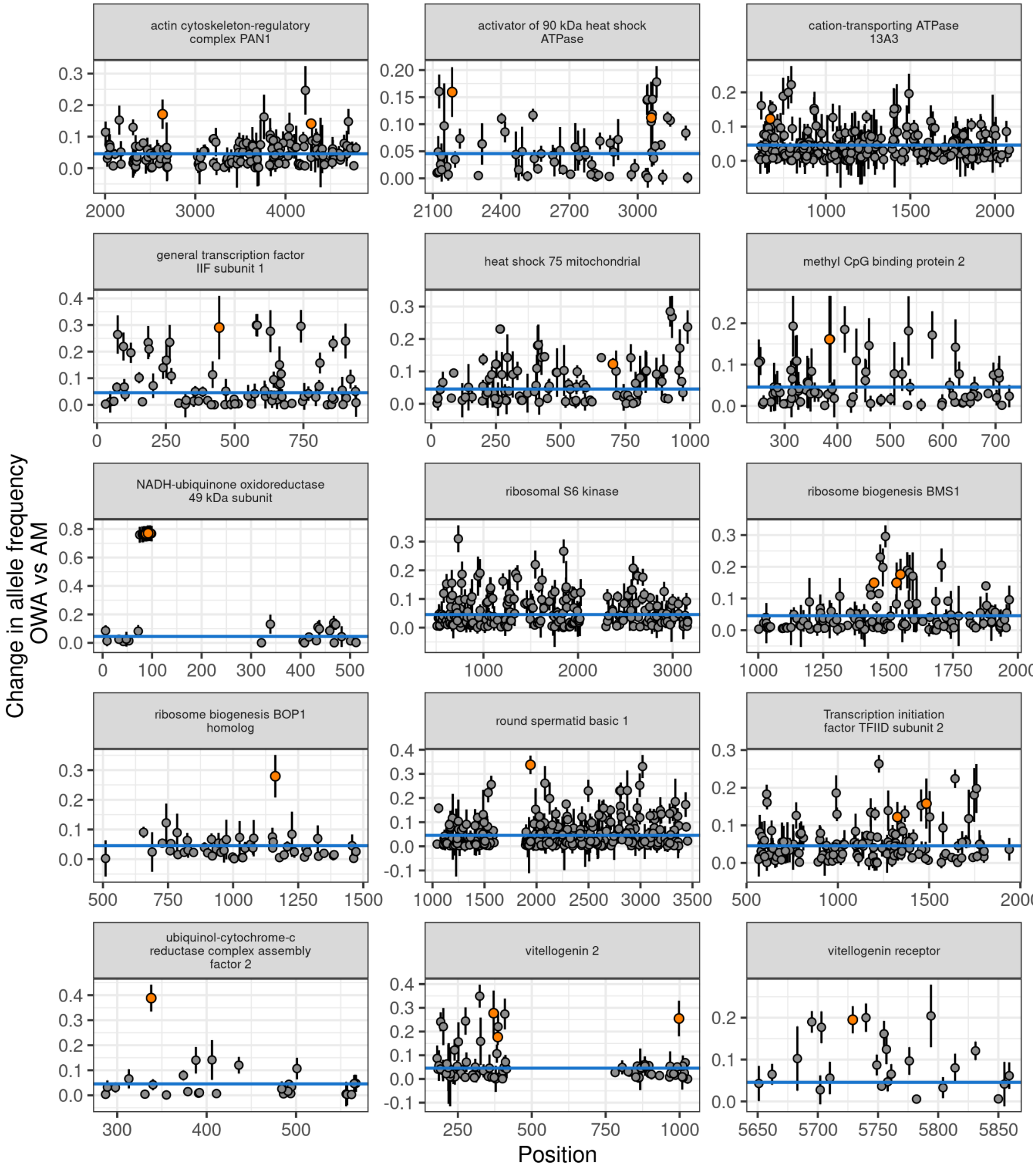

Figure S2: Candidate genes underlying adaptation to OWA environments. Points represent the average absolute change in allele frequency between AM_{AM} and OWA_{OWA} for F1, F2, and F3. Orange points are non-synonymous polymorphisms in the top 10% of allele frequency change distribution. Data are presented as mean values with standard error where n=4 independent replicates. Note that y-axes are different between plots.

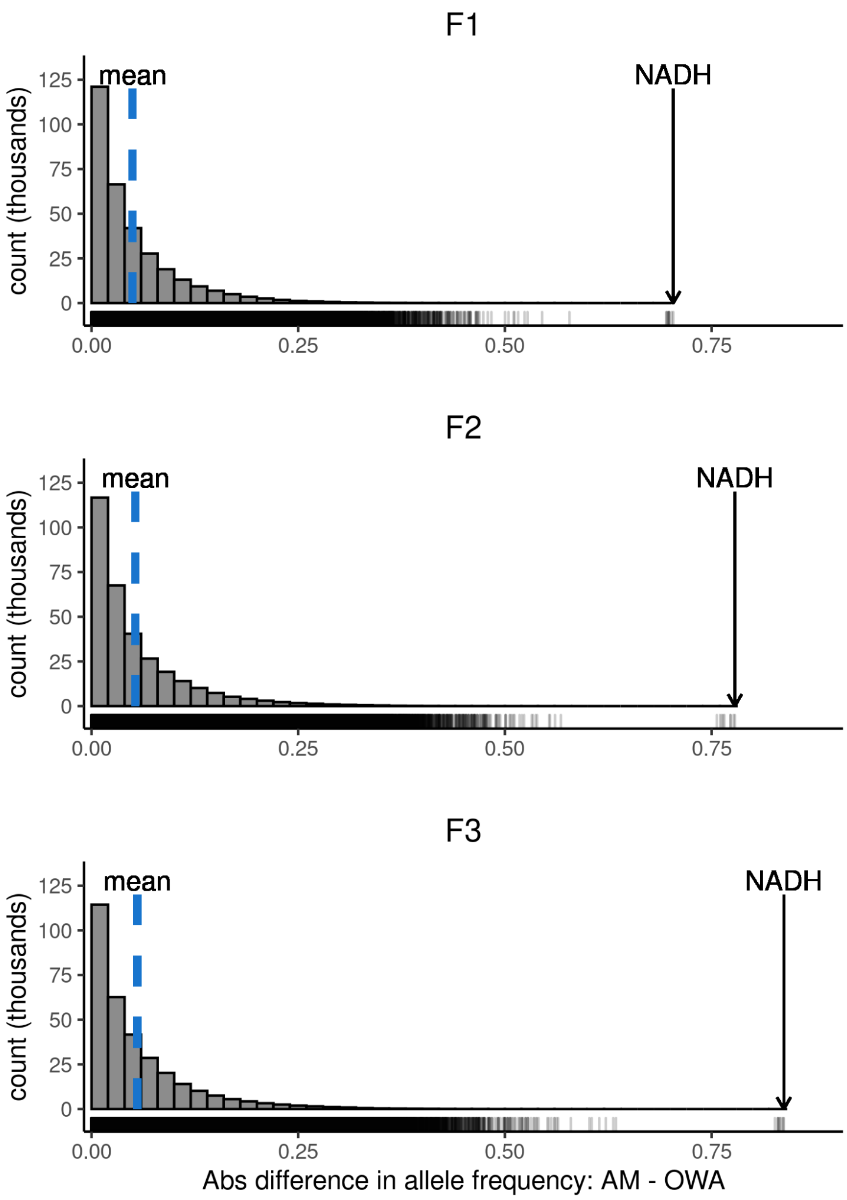

Figure S3: Change in allele frequencies between AM_{AM} and OWA_{OWA} for all three generations. The blue dashed line indicates the mean frequency change across all loci and the arrow shows the extreme frequency change of NADH-ubiquinone oxidoreductase 49 kDa subunit (NDUFS2) at each generation.

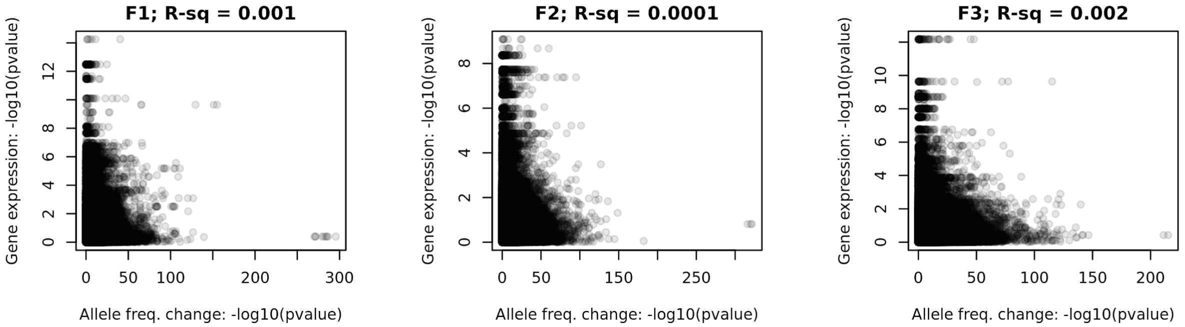

Figure S4: Relationship between gene expression and allele frequency divergence. X-axis is the -log10 of p-values from the CMH between AM and OWA in their home conditions. Y-axis corresponds to the -log10 of p-values of expression differences between AM and OWA in home conditions calculated with DESeq2 with the model ~Line + Treatment + Line:Treatment. For all generations, the variance explained is low, indicating that gene expression and allele frequency divergence is largely distinct.

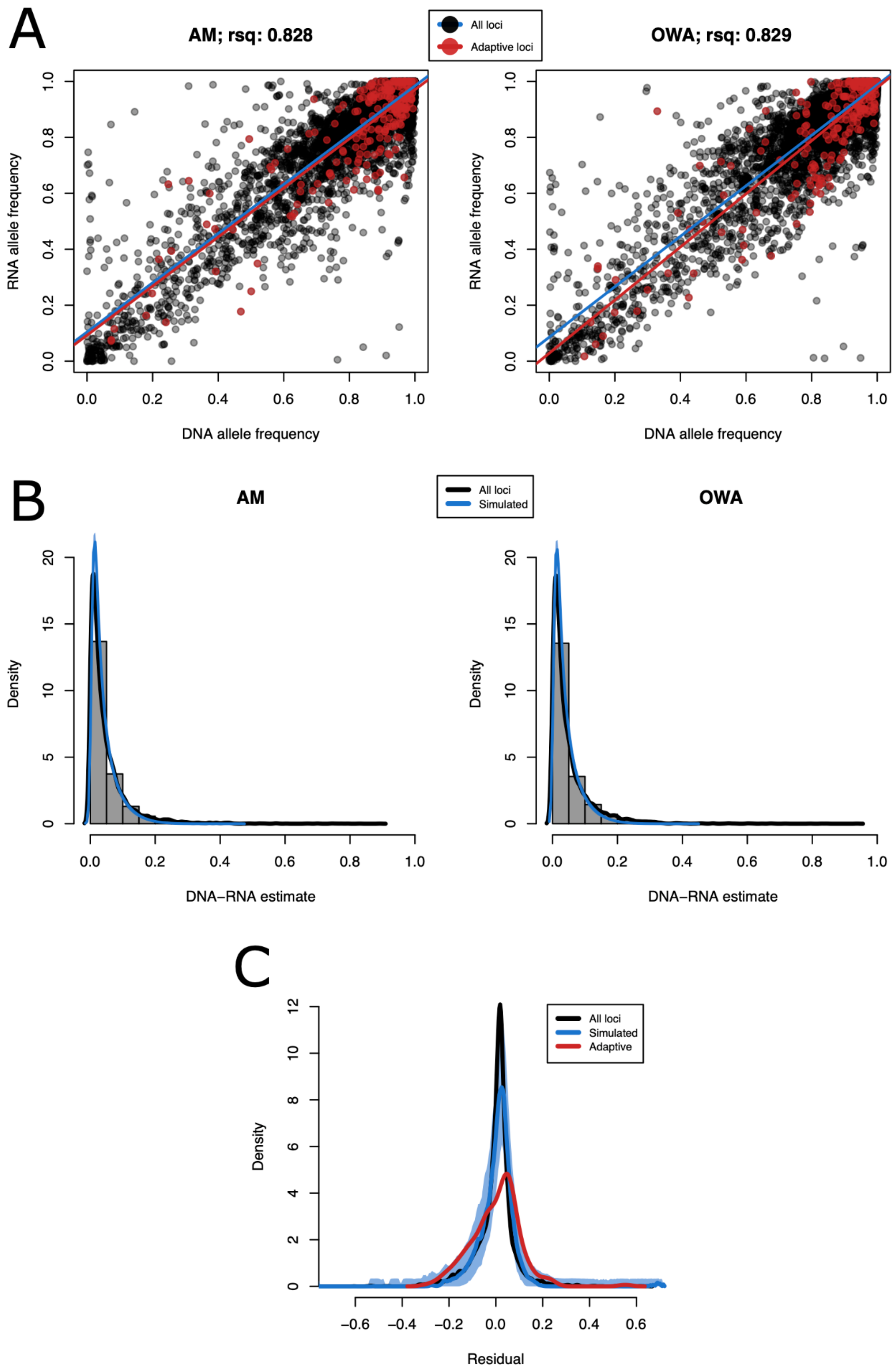

Figure S5: Comparison of allele frequency estimates from RNA and DNA for both OWA and AM. A). Each point is an overlapping locus where black indicates non-adaptive loci (as defined

in the main text) and red indicates adaptive loci. The blue line is the relationship between the two estimates for all loci, the red line for adaptive loci only. B) Comparing the absolute differences in DNA vs. RNA estimates for our observed data (histogram and black density plot) and for simulated data (blue line). Data were simulated 2,000 times under the Wright-Fisher model for two generations of drift and technical variance from pooling individuals for sequencing and finite sequencing depth. Simulated and observed data were highly congruent. This indicates that allele frequency estimates from RNA are consistent with those from DNA and that allele specific expression did not drive the results. C) Comparison of residual distance from A of this figure. Black indicates all loci and red indicates adaptive loci. Blue line is the median while shading represents the 95% confidence interval from 2000 datasets randomly drawn from all loci. These samples matched the number of adaptive loci (228) and their allele frequency. The CI of these random samples overlaps with the adaptive loci, indicating that the variance in the differences between DNA and RNA allele frequency estimates for adaptive loci is the same as those from all loci.

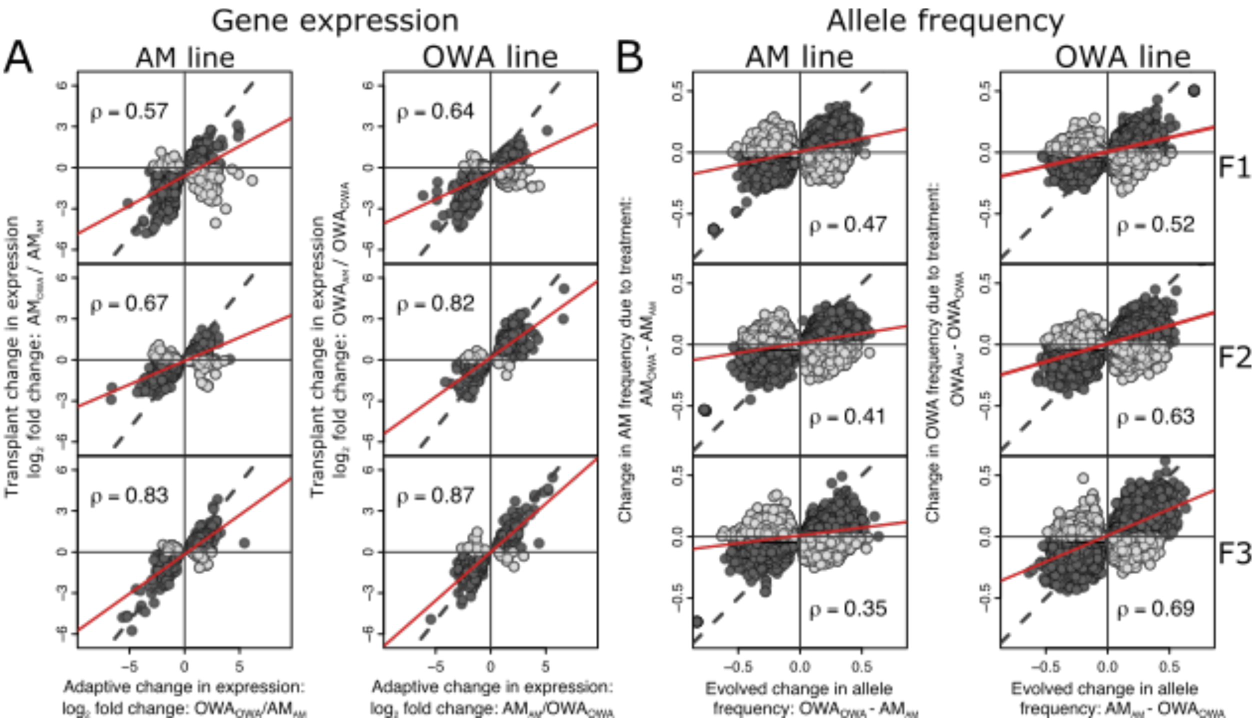

Figure S6: Convergence on evolved differences following transplant. For all plots, the dashed black line is a 1:1 relationship, the solid red line is the observed relationship between the x and y axes. The x-axis is the evolved and adaptive differences between the lines in their home environment. The y-axis compares each line in its transplanted environment to its home environment. Plots illustrate whether transplanted lines converge on the adaptive pattern for a given treatment condition. Dark points show the same pattern as the adaptive difference, light points are in the opposite direction. Pearson's correlation (ρ) is presented for each plot at each generation; generations organized F1 to F3, top to bottom. (A) Evolved changes in gene expression between OWA and AM in their home environments (OWA_{OWA} and AM_{AM}; x-axis) versus the transplant changes in expression (OWA_{AM} and AM_{OWA}; y-axis) for the same genes. (B) Evolved differences in allele frequency between OWA and AM in their home environment (x-axis) versus the change in allele frequency after transplant (y-axis) for the same genes.

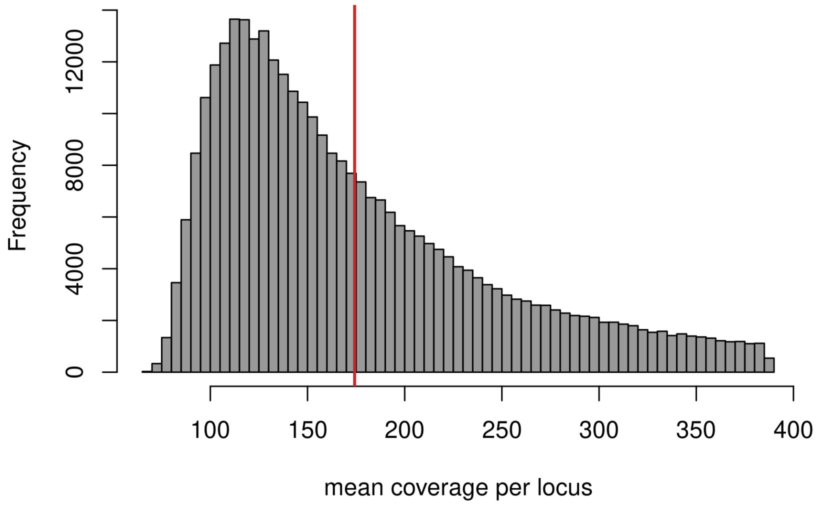

Figure S7: Histogram of mean coverage per locus. The vertical red line shows the mean coverage of 174x. Median coverage is 151x, and only 9% of loci have mean coverage < 100x.

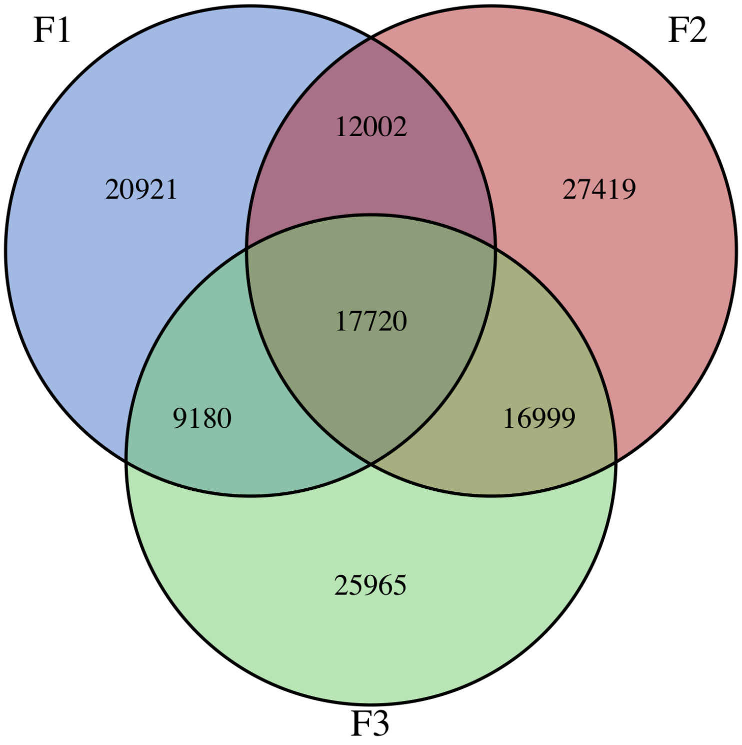

Figure S8: Significant loci ($P < 5.17e\text{-}08$) from two-sided CMH tests for each generation between OWA_{OWA} and AM_{AM}. Loci significant in all three generations were considered targets of selection and adaptive.

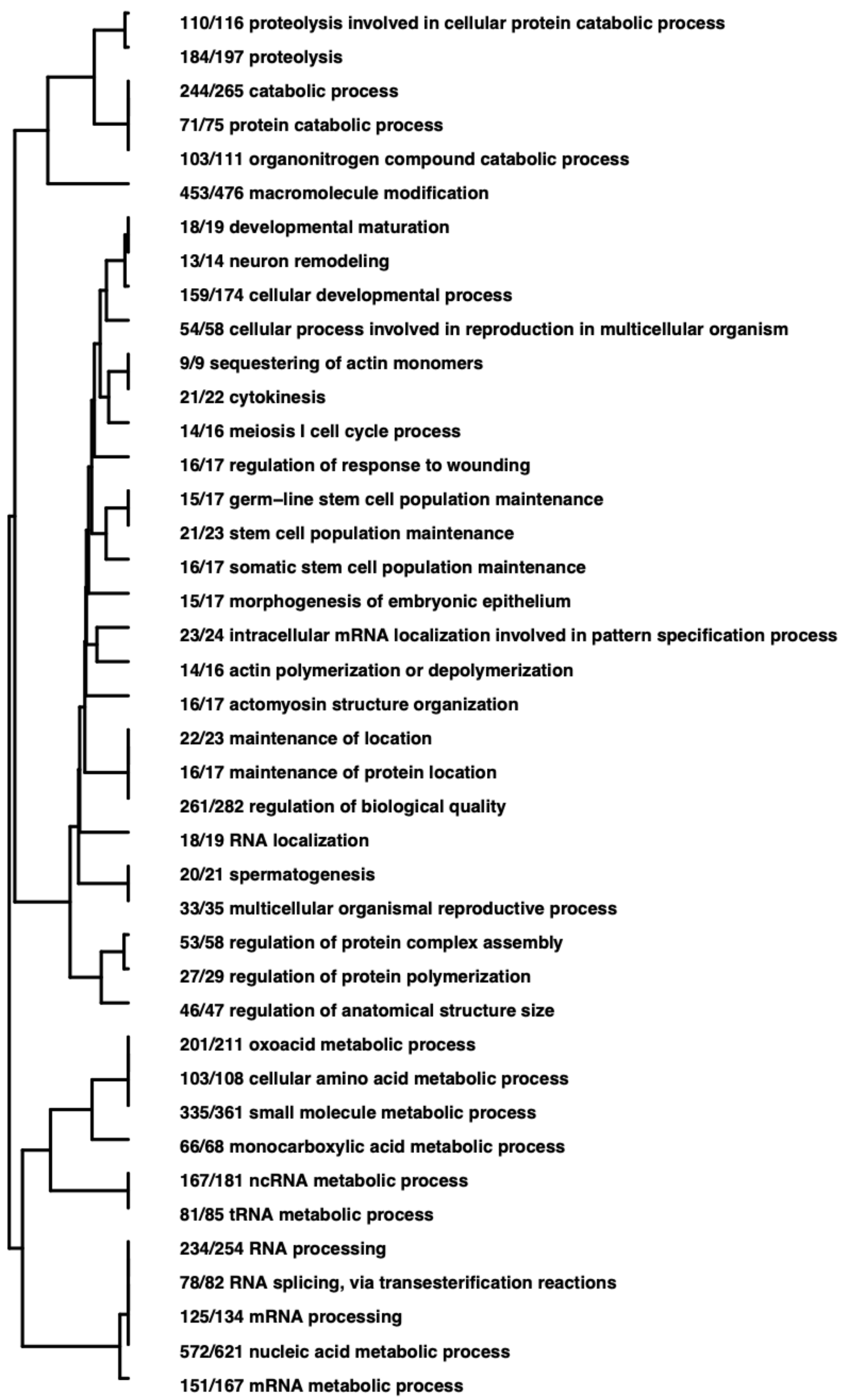

Figure S9: Gene ontology enrichment ($P < 0.05$) for allele frequency divergence between AM and OWA after 20 generations of selection. Results from one-sided tests in GO Mann-Whitney U. This tree represents the full enrichment set in figure 2C.

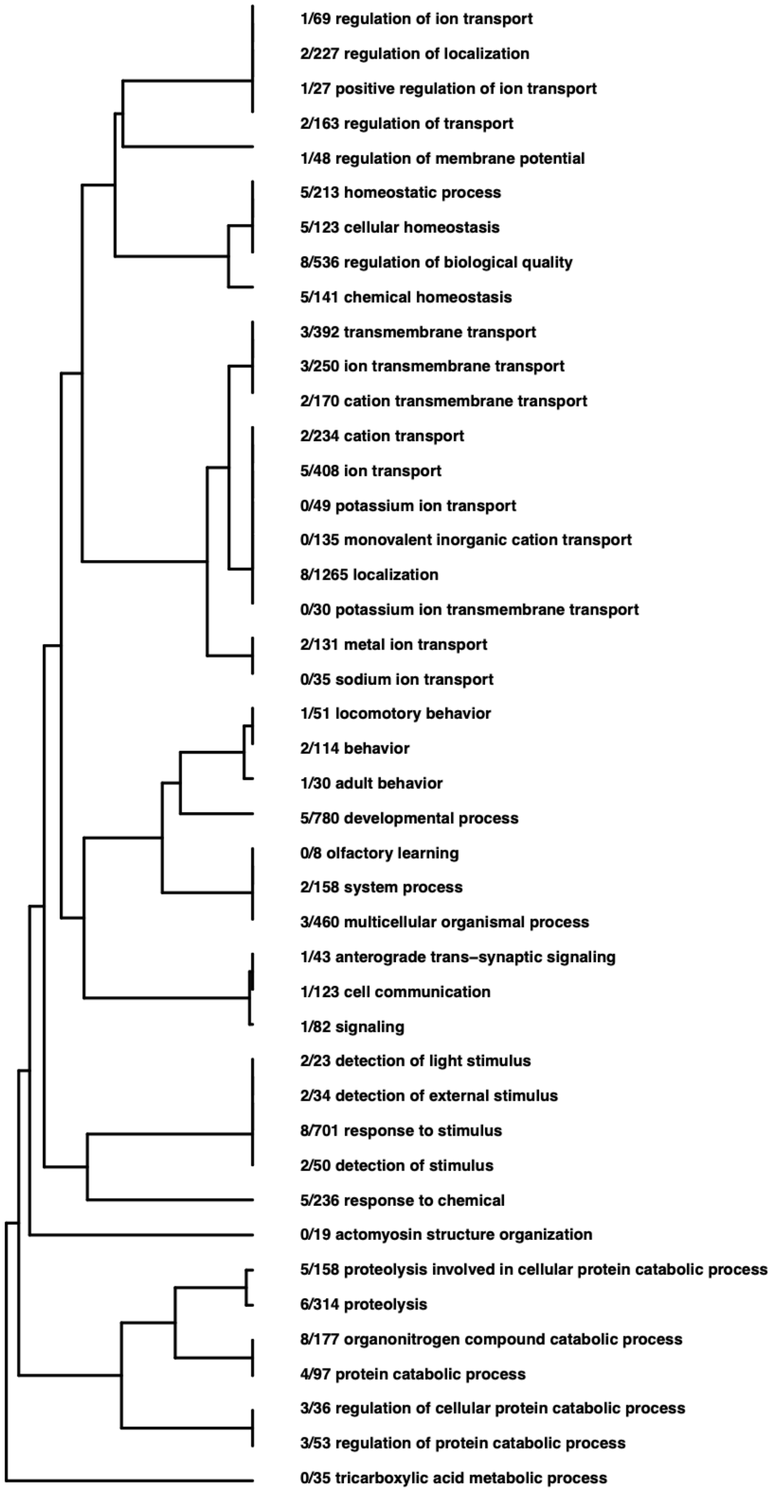

Figure S10: Gene ontology enrichment ($P < 0.05$) for gene expression divergence between AM and OWA after 20 generations of selection. Results from one-sided tests in GO Mann-Whitney U. This tree represents the full enrichment set in figure 2D.

Supplementary References

1. Dam, H. G. *et al.* Rapid, but limited, zooplankton adaptation to simultaneous warming and acidification. *Nat. Clim. Chang.* **11**, 780–786 (2021).
2. Rogier, O. *et al.* Accuracy of RNAseq based SNP discovery and genotyping in Populus nigra. *BMC Genomics* **19**, 1–12 (2018).
3. Konczal, M., Koteja, P., Stuglik, M. T., Radwan, J. & Babik, W. Accuracy of allele frequency estimation using pooled RNA-Seq. *Mol. Ecol. Resour.* **14**, 381–392 (2014).
4. Tangwancharoen, S., Semmens, B. X. & Burton, R. S. Allele-Specific Expression and Evolution of Gene Regulation Underlying Acute Heat Stress Response and Local Adaptation in the Copepod Tigriopus californicus. *J. Hered.* **111**, 539–547 (2020).
5. Serre, D. *et al.* Differential allelic expression in the human genome: a robust approach to identify genetic and epigenetic cis-acting mechanisms regulating gene expression. *PLoS Genet.* **4**, e1000006 (2008).
6. Ge, B. *et al.* Global patterns of cis variation in human cells revealed by high-density allelic expression analysis. *Nat. Genet.* **41**, 1216–1222 (2009).
7. Bell, G. D. M., Kane, N. C., Rieseberg, L. H. & Adams, K. L. RNA-Seq Analysis of Allele-Specific Expression, Hybrid Effects, and Regulatory Divergence in Hybrids Compared with Their Parents from Natural Populations. *Genome Biol. Evol.* **5**, 1309–1323 (2013).
8. Schlötterer, C., Tobler, R., Kofler, R. & Nolte, V. Sequencing pools of individuals - mining genome-wide polymorphism data without big funding. *Nat. Rev. Genet.* **15**, 749–763 (2014).
9. Li, L. *et al.* Divergence and plasticity shape adaptive potential of the Pacific oyster. *Nature Ecology & Evolution* 1 (2018).
10. Mallard, F., Jakšić, A. M. & Schlötterer, C. Contesting the evidence for non-adaptive

plasticity. *Nature* **555**, E21–E22 (2018).

11. Ho, W.-C. & Zhang, J. Genetic Gene Expression Changes during Environmental Adaptations Tend to Reverse Plastic Changes Even after the Correction for Statistical Nonindependence. *Mol. Biol. Evol.* **36**, 604–612 (2019).
12. Hurlbert, S. H. Pseudoreplication and the Design of Ecological Field Experiments. *Ecol. Monogr.* **54**, 187–211 (1984).
13. Schank, J. C. & Koehnle, T. J. Pseudoreplication is a pseudoproblem. *J. Comp. Psychol.* **123**, 421–433 (2009).
14. Oksanen, L. Logic of experiments in ecology: is pseudoreplication a pseudoissue? *Oikos* **94**, 27–38 (2001).
15. Sasaki, M. C. & Dam, H. G. Integrating patterns of thermal tolerance and phenotypic plasticity with population genetics to improve understanding of vulnerability to warming in a widespread copepod. *Glob. Chang. Biol.* **25**, 4147–4164 (2019).
