# Supplementary material for: Loss of transcriptional plasticity but sustained adaptive capacity after adaptation to global change conditions in a marine copepod
Source: Nat Commun. 2022 Mar 3;13:1147. doi: 10.1038/s41467-022-28742-6 (PMC8894427; doi:10.1038/s41467-022-28742-6)
Supplement: Supplementary file 2 — Description of Additional Supplementary Files [file 41467_2022_28742_MOESM2_ESM.docx]

Description of Additional Supplementary Files

Title: Supplementary Data 1

Description: Summary table of differential gene expression. AAAA = ambient line in ambient conditions; HHHH = warming and acidification in warming and acidification conditions; HHAA = ambient in warming and acidification conditions; AAHH = warming and acidification in ambient conditions. DAPC results are the contribution score for each gene for the DAPC loading. P-values and fold change were calculated with DESeq2.

Title: Supplementary Data 2

Description: Gene ontology enrichment results for gene expression.

Title: Supplementary Data 3

Description: Gene ontology enrichment results allele frequency data

Title: Supplementary Data 4

Description: Gene ontology enrichment for the change in genetic diversity following transplant of OWA to AM conditions.
