# Supplementary material for: Loss of transcriptional plasticity but sustained adaptive capacity after adaptation to global change conditions in a marine copepod
Source: Nat Commun. 2022 Mar 3;13:1147. doi: 10.1038/s41467-022-28742-6 (PMC8894427; doi:10.1038/s41467-022-28742-6)
Supplement: Supplementary file 7 — Reporting Summary [file 41467_2022_28742_MOESM7_ESM.pdf]

nature portfolio

Corresponding author(s): Reid Brennan

Last updated by author(s): Jan 12, 2022

Reporting Summary

Nature Portfolio wishes to improve the reproducibility of the work that we publish. This form provides structure for consistency and transparency in reporting. For further information on Nature Portfolio policies, see our Editorial Policies and the Editorial Policy Checklist.

Statistics

For all statistical analyses, confirm that the following items are present in the figure legend, table legend, main text, or Methods section.

n/a	Confirmed	
☐	☒	The exact sample size (n) for each experimental group/condition, given as a discrete number and unit of measurement
☐	☒	A statement on whether measurements were taken from distinct samples or whether the same sample was measured repeatedly
☐	☒	The statistical test(s) used AND whether they are one- or two-sided<br>*Only common tests should be described solely by name; describe more complex techniques in the Methods section.*
☐	☒	A description of all covariates tested
☐	☒	A description of any assumptions or corrections, such as tests of normality and adjustment for multiple comparisons
☐	☒	A full description of the statistical parameters including central tendency (e.g. means) or other basic estimates (e.g. regression coefficient) AND variation (e.g. standard deviation) or associated estimates of uncertainty (e.g. confidence intervals)
☐	☒	For null hypothesis testing, the test statistic (e.g. F, t, r) with confidence intervals, effect sizes, degrees of freedom and P value noted<br>*Give P values as exact values whenever suitable.*
☐	☒	For Bayesian analysis, information on the choice of priors and Markov chain Monte Carlo settings
☐	☒	For hierarchical and complex designs, identification of the appropriate level for tests and full reporting of outcomes
☐	☒	Estimates of effect sizes (e.g. Cohen's d, Pearson's r), indicating how they were calculated

Our web collection on statistics for biologists contains articles on many of the points above.

Software and code

Policy information about availability of computer code

Data collection	No software was used
Data analysis	All analyses were run in R version 3.6.0. Code to reproduce all analyses are archived on Zenodo: doi.org/10.5281/zenodo.5840148

For manuscripts utilizing custom algorithms or software that are central to the research but not yet described in published literature, software must be made available to editors and reviewers. We strongly encourage code deposition in a community repository (e.g. GitHub). See the Nature Portfolio guidelines for submitting code & software for further information.

Data

Policy information about availability of data

All manuscripts must include a data availability statement. This statement should provide the following information, where applicable:

- Accession codes, unique identifiers, or web links for publicly available datasets
- A description of any restrictions on data availability
- For clinical datasets or third party data, please ensure that the statement adheres to our policy

The raw sequence data generated in this study have been deposited at NCBI under BioProject PRJNA555881. Life history data are available as a supplemental file and allele frequency and gene expression table is available on Figshare (https://doi.org/10.6084/m9.figshare.10301690).

Field-specific reporting

Please select the one below that is the best fit for your research. If you are not sure, read the appropriate sections before making your selection.

☐ Life sciences ☐ Behavioural & social sciences ☒ Ecological, evolutionary & environmental sciences

For a reference copy of the document with all sections, see nature.com/documents/nr-reporting-summary-flat.pdf

Ecological, evolutionary & environmental sciences study design

All studies must disclose on these points even when the disclosure is negative.

Study description

This experimental evolution study was designed based on previous simulation work to determine replicate number, sample size, and number of generations. We included 4 replicates for each condition for the initial experimental evolution. The starting population consisted of 2000 adults that were used to generate a lab acclimated population (3 generations) and seed each replicate treatment. Population sizes were kept in the thousands throughout the experiment and experimental evolution was conducted for 20 generations. These parameters follow established statistical recommendations (see references below). For the reciprocal transplant, 4 replicates are sufficient to identify differential expression.

Kofler, R. and Schlötterer, C., 2014. A guide for the design of evolve and resequencing studies. Molecular biology and evolution, 31 (2), pp.474-483.

Baldwin-Brown, J.G., Long, A.D. and Thornton, K.R., 2014. The power to detect quantitative trait loci using resequenced, experimentally evolved populations of diploid, sexual organisms. Molecular biology and evolution, 31(4), pp.1040-1055.

Research sample

2000 adult Acartia tonsa of mixed sex were collected from Esker Point Beach in Groton, CT, USA, decimal degrees: 41.320725, -72.001643. This sample represents a population from the middle of the coastal distribution of A. tonsa in the US, though this is a globally distributed species. This sample size is much larger than the minimum starting haplotype number of 500 suggested by Baldwin-Brown et al.

Baldwin-Brown, J.G., Long, A.D. and Thornton, K.R., 2014. The power to detect quantitative trait loci using resequenced, experimentally evolved populations of diploid, sexual organisms. Molecular biology and evolution, 31(4), pp.1040-1055.

Sampling strategy

4 replicates were used per treatment, based on simulations in the following manuscripts. The experiment was completed one time.

Kofler, R. and Schlötterer, C., 2014. A guide for the design of evolve and resequencing studies. Molecular biology and evolution, 31 (2), pp.474-483.

Baldwin-Brown, J.G., Long, A.D. and Thornton, K.R., 2014. The power to detect quantitative trait loci using resequenced, experimentally evolved populations of diploid, sexual organisms. Molecular biology and evolution, 31(4), pp.1040-1055.

Data collection

Fitness data were collected by JAD and recorded as experiments were completed with no additional instruments. Sequence data was generated by Novogene on an Illumina NovaSeq 600.

Timing and spatial scale

Spatial scale is a single population collected from Groton CT. The experiment was run for 25 generations with 3 generations of reciprocal transplant. Data were collected at each transplant generation with no gap.

Data exclusions

No data were excluded from the analysis

Reproducibility

Due to the time and logistical constraints of experimental evolution, the experiment could only be completed once. However, we include four successful replicates to assure repeatability of our findings.

Randomization

Individuals seeded to each replicate treatment were chosen at random from the founding population. Eggs were randomly allocated to each treatment to achieve identical starting density.

Blinding

Blinding was not possible due to personnel constraints. Because all feeding and data were quantitative, lack of blinding should not skew results.

Did the study involve field work? ☐ Yes ☒ No

Reporting for specific materials, systems and methods

We require information from authors about some types of materials, experimental systems and methods used in many studies. Here, indicate whether each material, system or method listed is relevant to your study. If you are not sure if a list item applies to your research, read the appropriate section before selecting a response.

Materials & experimental systems

n/a	Involved in the study
☒	☐ Antibodies
☒	☐ Eukaryotic cell lines
☒	☐ Palaeontology and archaeology
☐	☒ Animals and other organisms
☒	☐ Human research participants
☒	☐ Clinical data
☒	☐ Dual use research of concern

Methods

n/a	Involved in the study
☒	☐ ChIP-seq
☒	☐ Flow cytometry
☒	☐ MRI-based neuroimaging

Animals and other organisms

Policy information about studies involving animals; ARRIVE guidelines recommended for reporting animal research

Laboratory animals	No laboratory animals were used
Wild animals	Acartia tonsa of mixed sex were collected as adults from the wild using plankton nets. They were transported in buckets with sea water. Cultures of animals are still being gown in the lab.
Field-collected samples	Wild, adult acartia tonsa were collected using a plankton net and transported in buckets to UConn Avery Point where they were maintained in common garden environments until the start of the experiment. At the end of the experiment, animals were euthanized due to limited space and resources. Animals were maintained in 3L culture containers at 18C and 12hL:12hD photoperiod. Cultures were fed Cultures were fed every 48-72 hours at food-replete concentrations of carbon (>800 µg C/L) split equally between three species of prey phytoplankton. Prey phytoplankton included Tetraselmis spp., Rhodomonas spp., and Thalassiosira weissflogii, which is a common diet combination used for rearing copepods. Culture water was changed weekly.
Ethics oversight	No ethical approval was needed due to work with invertebrate copepods.

Note that full information on the approval of the study protocol must also be provided in the manuscript.
